# Supplementary material for: Novel Driver Strength Index highlights important cancer genes in TCGA PanCanAtlas patients
Source: PeerJ. 2022 Aug 11;10:e13860. doi: 10.7717/peerj.13860 (PMC9375969; doi:10.7717/peerj.13860)
Supplement: Supplemental Information 2 [file peerj-10-13860-s002.zip › COHORTS/genes plots/stage/2021_8_16_14_9_distribution_stage_females_III.pdf]

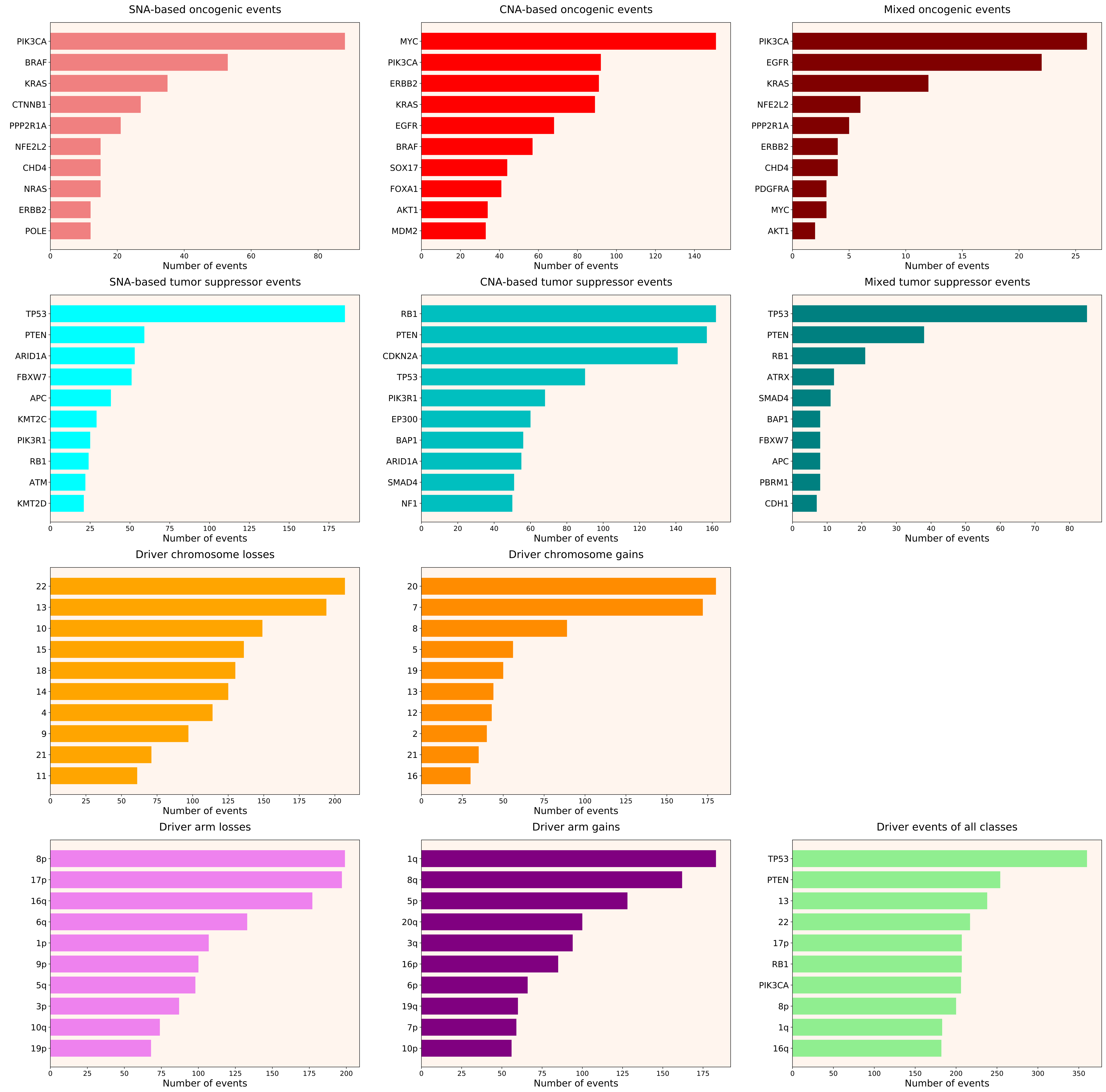
SNA-based oncogenic events
PIK3CA
BRAF
KRAS
CTNNB1
PPP2R1A
NFE2L2
CHD4
NRAS
ERBB2
POLE
Number of events
CNA-based oncogenic events
MYC
PIK3CA
ERBB2
KRAS
EGFR
BRAF
SOX17
FOXA1
AKT1
MDM2
Number of events
Mixed oncogenic events
PIK3CA
EGFR
KRAS
NFE2L2
PPP2R1A
ERBB2
CHD4
PDGFRA
MYC
AKT1
Number of events
SNA-based tumor suppressor events
TP53
PTEN
ARID1A
FBXW7
APC
KMT2C
PIK3R1
RB1
ATM
KMT2D
Number of events
CNA-based tumor suppressor events
RB1
PTEN
CDKN2A
TP53
PIK3R1
EP300
BAP1
ARID1A
SMAD4
NF1
Number of events
Mixed tumor suppressor events
TP53
PTEN
RB1
ATRX
SMAD4
BAP1
FBXW7
APC
PBRM1
CDH1
Number of events
Driver chromosome losses
22
13
10
15
18
14
4
9
21
11
Number of events
Driver chromosome gains
20
7
8
5
19
13
12
2
21
16
Number of events
Driver arm losses
8p
17p
16q
6q
1p
9p
5q
3p
10q
19p
Number of events
Driver arm gains
1q
8q
5p
20q
3q
16p
6p
19q
7p
10p
Number of events
Driver events of all classes
TP53
PTEN
13
22
17p
RB1
PIK3CA
8p
1q
16q
Number of events
